# Supplementary material for: Clinical Features and Management of Acute and Chronic Radiation-Induced Colitis and Proctopathy
Source: Cancers (Basel). 2023 Jun 12;15(12):3160. doi: 10.3390/cancers15123160 (PMC10296205; doi:10.3390/cancers15123160)
Supplement: Supplementary file 1 [file cancers-15-03160-s001.zip › cancers-2390140-supplementary.pdf]

Supplementary Table S1. Univariate analyses

Covariate	Odds ratio (95% confidence interval)	*P* value
**Endoscopically apparent bleeding**		
Age	1.02 (0.99-1.05)	0.267
Female vs. male	0.38 (0.17-0.83)	0.015
NSAID	0.86 (0.35-2.13)	0.740
Hypertension	1.07 (0.49-2.31)	0.867
Cardiac diseases	1.80 (0.52-6.24)	0.354
Diabetes	2.54 (0.76-8.43)	0.128
GI-toxic chemotherapy	0.18 (0.08-0.41)	<0.001
Length of radiotherapy	1.04 (1.00-1.09)	0.032
Dose of radiotherapy	1.04 (1.01-1.08)	0.013
Number of radiotherapy sessions	1.05 (0.99-1.12)	0.112
Radiotherapy type[a]	1.96 (0.69-5.55)	0.205
CRICAP vs. ARICAP onset	3.41 (1.56-7.47)	0.002
Medical treatment	0.49 (0.21-1.11)	0.086
**Need for medical treatment**		
Age	0.97 (0.94-1.00)	0.060
Female vs male	3.16 (1.40-7.14)	0.006
NSAID	4.62 (1.28-16.66)	0.019
GI-toxic chemotherapy	3.18 (1.39-7.26)	0.006
Length of radiotherapy	0.97 (0.93-1.01)	0.092
Dose of radiotherapy	0.94 (0.91-0.97)	<0.001
Number of radiotherapy sessions	0.88 (0.82-0.95)	0.001
Radiotherapy type[a]	1.24 (0.44-3.55)	0.683
CRICAP vs. ARICAP onset	0.15 (0.06 -0.40)	<0.001
**Need for endoscopic APC treatment**		
Age	1.10 (1.05-1.15)	<0.001
Female vs. male	0.11 (0.04-0.28)	<0.001
NSAID	1.90 (0.75-4.86)	0.178
GI-toxic chemotherapy	0.29 (0.12-0.69)	0.005
Radiotherapy type[a]	0.21 (0.05-0.95)	0.043
Length of radiotherapy	1.08 (1.03-1.13)	0.002
Dose of radiotherapy	1.08 (1.04-1.12)	<0.001
Number of radiotherapy sessions	1.12 (1.04-1.20)	0.002
Requirement of medical treatment	1.11 (0.47-2.61)	0.816
Duration of medical treatment	1.02 (0.99-1.05)	0.220
Symptoms of RICAP		
Bleeding	2.65 (1.07-6.57)	0.036
Diarrhea	0.35 (0.14-0.87)	0.024
Abdominal pain	0.24 (0.08-0.75)	0.014
Duration of symptoms	1.01 (1.00-1.01)	0.071
CRICAP vs. ARICAP onset	3.99 (1.61-9.91)	0.003
Early vs. delayed endoscopy	0.72 (0.30-1.74)	0.466

Recurrent RICAP symptoms before endoscopy	1.21 (0.49-2.96)	0.679
**Recurrence of RICAP symptoms**		
Age	0.98 (0.95-1.01)	0.213
Female vs. male	0.58 (0.26-1.28)	0.175
NSAID	2.39 (0.82-7.01)	0.112
GI-toxic chemotherapy	0.57 (0.26-1.26)	0.167
Radiotherapy type[a]	1.76 (0.59-5.27)	0.313
Length of radiotherapy	0.98 (0.94-1.02)	0.246
Dose of radiotherapy	0.99 (0.96-1.02)	0.660
Number of radiotherapy sessions	1.00 (0.94-1.06)	0.976
Requirement of medical treatment	1.36 (0.60-3.06)	0.460
Duration of medical treatment	1.04 (1.00-1.08)	0.072
ARICAP vs. CRICAP onset	1.67 (0.76-3.66)	0.198
Early endoscopy vs. delayed endoscopy	0.45 (0.17-1.16)	0.097

[a]Brachytherapy vs external beam.

Abbreviations: APC, argon plasma coagulation; ARICAP, acute radiation-induced colitis and proctopathy; CRICAP, chronic radiation-induced colitis and proctopathy; GI, gastrointestinal; NSAID, nonsteroidal anti-inflammatory drug; RICAP, radiation-induced colitis and proctopathy.

Supplementary Table S2. Clinical characteristics and outcomes in patients stratified by the presence of luminal ulceration and location of ARICAP on endoscopic evaluation

Covariate	Ulcers<br>N = 8	No ulcers<br>N = 43	*P* value	Proctitis<br>N = 45	Non-Rectal ARICAP<br>N = 6	*P* value
Median length of radiotherapy, days (IQR) (N = 51)	40 (35-44)	41 (37-42)	0.873	41 (37-43)	41 (33-47)	0.948
Median dose of radiotherapy, Gy (IQR)	55 (47 -57)	53 (45-58)	0.649	54 (45-58)	52 (44-59)	0.769
Median number of radiotherapy sessions (IQR)	30 (27-30)	28 (25-30)	0.415	29 (25-30)	28 (25-31)	0.840
Median time from radiotherapy to RICAP, days (IQR) (N = 51)	10 (3-20)	15 (7-21)	0.303	15 (8-21)	10 (4-26)	0.478
Median duration of symptoms, days (IQR)(N = 48)	20 (16-28)	15 (8-23)	0.322	16 (8-23)	11 (6-41)	0.379
Median duration of hospitalization, days (IQR) (N = 9)	4 (4-NA)	4 (2-10)	0.655	4 (2-17)	5 (2-NA)	0.889
GI-toxic chemotherapy (%)	7 (88)	30 (70)	0.419	34 (76)	3 (50)	0.327
Diarrhea grade[a] (N = 42) (%)			0.178			0.151
1	1 (17)	11 (30)		9 (24)	3 (60)	
2	2 (33)	20 (54)		21 (55)	1 (20)	
3	3 (50)	6 (16)		8 (21)	1 (20)	
Colitis grade[a] (N = 50) (%)			0.184			0.086
1	0 (0)	10 (24)		7 (15.9)	3 (50)	
2-3	8 (100)	32 (76)		37 (84)	3 (50)	
Medical treatment	8 (100)	36 (84)	0.579	39 (87)	5 (83)	1.000
Median duration of medical treatment, days (IQR) (N = 41)	25 (17-32)	15 (8-23)	0.076	18 (10-28)	7 (5-13)	0.032
Repeat APC treatment (%)	0 (0)	3 (7)	1.000	2 (4)	1 (17)	0.319
RICAP-associated strictures (%)	0 (0)	1 (2)	1.000	1 (2)	0 (0)	1.000
Median duration between initial RICAP onset and initial endoscopy, days (IQR) (N = 51)	144 (97-470)	567 (200-1841)	0.005	389 (163-992)	722 (437-2397)	0.278
Recurrence of RICAP after endoscopy (%)	4 (50)	7 (16)	0.055	10 (22)	1 (17)	1.000

Abbreviations: APC, argon plasma coagulation; ARICAP, acute radiation-induced colitis and proctopathy; GI, gastrointestinal; IQR, interquartile range; RICAP, radiation-induced colitis and proctopathy.

[a] % is calculated based on the number of patients with available information of diarrhea or colitis grade.

Supplementary Table S3. Clinical characteristics and outcomes in patients stratified by the presence of luminal ulceration and location of CRICAP on endoscopic evaluation.

Covariate	Ulcers N = 12	No ulcers N = 49	*P* value	Proctitis N = 53	Non-Rectal CRICAP N = 8	*P* value
Median length of radiotherapy, days (IQR) (N = 61)	45 (39-55)	49 (35-55)	0.819	50 (39-56)	33 (20-36)	0.002
Median dose of radiotherapy, Gy (IQR)	58 (50-74)	70 (51-76)	0.656	70 (54-76)	45 (44-52)	0.001
Median number of radiotherapy sessions (IQR)	33 (29-37)	35 (28-38)	0.882	35 (29-38)	25 (24-34)	0.034
Median time from radiotherapy to RICAP, days (IQR) (N = 61)	340 (145-1046)	413 (199-649)	0.933	404 (216-613)	548 (36-1226)	0.990
Median duration of symptoms, days (IQR) (N = 58)	27 (17-158)	29 (14-66)	0.411	30 (21-73)	14 (8-180)	0.326
Median duration of hospitalization, days (IQR) (N = 7)	5 (3-N/A)	5 (2-9)	0.244	5 (2-10)	6 (3-N/A)	0.422
GI system-toxic chemotherapy (%)	5 (42%)	14 (29)	0.489	15 (28)	4 (50)	0.241
Diarrhea grade[a] (N = 21) (%)			1.000			0.119
1	1 (25)	6 (35)		5 (29)	2 (50)	
2	2 (50)	7 (41)		9 (53)	0 (0)	
3	1 (25)	4 (24)		3 (18)	2 (50)	
Colitis grade (%)			1.000			1.000
1	0 (0)	1 (2)		1 (2)	0 (0)	
2-3	12 (100)	48 (98)		52 (98)	8 (100)	
Medical treatment (%)	6 (50)	24 (49)	1.000	26 (49)	4 (50)	1.000
Mean duration of medical treatment, days (SD) (N = 22)	28 (14-37)	19 (10-36)	0.639	26 (10-36)	14 (9-34)	0.524
Repeat APC treatment (%)	3 (25)	9 (18)	0.689	12 (23)	0 (0)	0.337
RICAP associated strictures (%)	0 (0)	3 (6)	1.000	1 (2)	2 (25)	0.043
Median duration between initial colitis onset and initial endoscopy, days (IQR) (N = 61)	158 (19-240)	52 (11-216)	0.307	65 (13-183)	146 (11-324)	0.403
Recurrence of RICAP after endoscopy (%)	3 (25)	30 (61)	0.049	30 (57)	3 (38)	0.451

Abbreviations: APC, argon plasma coagulation; CRICAP, chronic radiation-induced colitis and proctopathy; GI, gastrointestinal; IQR, interquartile range; N/A, not applicable; RICAP, radiation-induced colitis and proctopathy; SD, standard deviation.

[a] % is calculated based on the number of patients with available information of diarrhea grade.

Supplementary Table S4. Disease courses and outcomes in patients with RICAP stratified by whether endoscopic APC treatment was received

**Covariate**	**APC treatment N = 34**	**No APC treatment N = 78**	***P* value**
Median duration from initial RICAP to endoscopy, months (IQR) (N = 112)	4 (1-10)	9 (2-25)	0.028
Indication of endoscopy (%)			
Diarrhea	2 (6)	12 (15)	0.221
Bleeding/mucus	31 (91)	33 (42)	<0.001
Abdominal pain	1 (3)	12 (15)	0.104
Endoscopic presentation (%)			
Neovascularization	17 (79)	56 (72)	0.486
Bleeding	14 (41)	15 (19)	0.020
Ulcer	6 (18)	14 (18)	1.000
Stricture	1 (3)	3 (4)	1.000
Colitis treatment received (%)			
Loperamide	6 (18)	38 (49)	0.003
Diphenoxylate/atropine	4 (12)	32 (41)	0.002
Suppository/enema	9 (27)	15 (19)	0.455
Topical corticosteroid	6 (18)	2 (3)	0.009
Intravenous fluid	2 (6)	11 (14)	0.337
Median length of medical treatment, days (IQR) (N = 63)	19 (7-40)	17 (9-29)	0.854
Median duration of symptoms, days (IQR) (N = 104)	4 (1-9)	21 (11-30)	0.079
Median duration of hospitalization, days (IQR) (N = 16)	5 (3-9)	4 (2-9)	0.650
Recurrence of RICAP post endoscopy (%)	17 (50)	27 (35)	0.144

Abbreviations: APC, argon plasma coagulation; IQR, interquartile range; RICAP, radiation-induced colitis and proctopathy.
